# Depressive symptoms are related to asthma control but not self-management among rural adolescents

**DOI:** 10.3389/falgy.2023.1271791

**Published:** 2024-01-11

**Authors:** Neha B. Patel, Amarilis Céspedes, Jianfang Liu, Jean-Marie Bruzzese

**Affiliations:** ^1^ Division of Pediatric Pulmonary, Columbia University Medical Center, New York, NY, United States; ^2^ Columbia University School of Nursing, New York, NY, United States

**Keywords:** adolescents, asthma, comorbid, depression, mental health, non-urban, pediatrics, rural

## Abstract

**Background:**

Depression, a relevant comorbidity with asthma, has been reported to be associated with asthma morbidity. Asthma self-management is essential to asthma control and may be negatively impacted by depression. We examined these associations in rural adolescents, a group with relatively high asthma morbidity and depressive symptoms, a population often ignored in asthma research.

**Methods:**

We used baseline data from a randomized trial of an asthma intervention for adolescents in rural South Carolina (*n *= 197). Adolescents completed the Center for Epidemiological Studies-Depression (CES-D), three indices of asthma self-management (the Asthma Prevention Index, the Asthma Management Index and the Asthma Self-Efficacy Index), and the Asthma Control Test (ACT). Poisson and linear regression tested associations between depression, self-management, and asthma control. The models controlled for demographic variables and included school as a fixed effect.

**Results:**

Most participants (mean age = 16.3 ± 1.2 years) self-identified as female (68.5%) and Black (62.43%). The mean CES-D score was 19.7 ± 10.3, with 61.4% of participants at risk for depression. The depressive symptoms were significantly related to asthma control [*β *= −0.085, 95% confidence interval (CI) = −0.14 to −0.03] but not to prevention [relative risk (RR) = 1.00, 95% CI = 0.99–1.01], management (RR = 1.00, 95% CI = 0.99–1.01), or self-efficacy (*β* = −0.002, 95% CI = −0.01 to 0.01),

**Conclusions:**

In this sample of rural adolescents, as depressive symptoms increased, asthma control declined. Depressive symptoms were not associated with asthma self-management, suggesting that the aspects of self-management we assessed are not an avenue by which depression impacts asthma control. Additional research is needed to further understand the relationship between depressive symptoms, asthma self-management, and control.

## 1 Introduction

Asthma is the most common chronic pediatric condition in the United States. Adolescents are at increased risk of asthma and asthma-related morbidities (1, 2). Asthma contributes to a high number of Emergency Department (ED) visits and hospitalizations (2–4), as well as school absences and poor school performance (5, 6).

Depressive symptoms are a relevant asthma-related comorbidity that has been reported to be associated with asthma morbidity in children and adolescents with asthma (7–9). Asthma self-management is an essential skill to achieve asthma control and reduce asthma morbidity (10), and it is impacted by depressive symptoms. A recent review found that depressive symptoms are associated with asthma self-management among adolescents, with some studies finding that depressive symptoms are associated with worse self-management and others with better self-management (11). Specifically, depressive symptoms have consistently been found to be associated with worse trigger management; one study found that adolescents with depressive symptoms were more likely to see a healthcare provider for asthma (11). In addition, some data suggest that treating depression in adolescents with elevated depressive symptoms can improve asthma medication adherence (12). Additional research is needed to understand the role of depressive symptoms and asthma self-management.

Rural adolescents are a population with a high prevalence of asthma (13) and asthma-related morbidities (14, 15). Studies examining asthma among rural and urban patients have shown differences in asthma management by healthcare providers. Compared with urban youth, rural patients are often prescribed fewer anti-inflammatory medications (16–18) and receive less asthma education (18). While urban and rural youth reported that the COVID-19 pandemic did not impact their asthma severity, urban youth had greater odds of changing how they manage their asthma during the pandemic (19). For example, a greater proportion of urban youth reported increasing their medication (27.0% vs. 3.3%, respectively) and monitoring symptoms (12.7% vs. 2.5%, respectively).

The prevalence of depressive symptoms in rural adolescents was found to be as high as 34% (20). However, to the best of our knowledge, no studies examining asthma outcomes and depressive symptoms have included rural adolescents. To address this gap, we tested whether depressive symptoms are associated with asthma self-management and asthma control among rural adolescents with asthma. We hypothesized that an increase in depressive symptoms would be associated with decreased asthma self-management and poor asthma control in a rural adolescent population.

## 2 Methods

### 2.1 Study design, sample, and procedure

This study is a secondary data analysis of baseline data from 197 adolescents participating in a randomized trial testing the effectiveness of a school-based intervention for adolescents with uncontrolled asthma in South Carolina. Schools (*n* = 8) were from communities defined as rural based on the Rural-Urban Commuting Area codes (21). The inclusion criteria for the clinical trial were being at least 13 years old, being diagnosed with asthma, and having signs and symptoms of asthma morbidity defined as follows: (1) in the past month: presenting daytime symptoms at least once a week, activity limitations at least once a week, night wakening at least two nights a month, or rescue inhaler use when symptomatic at least once a week; or (2) in the past 12 months: one or more asthma-related urgent visits to a medical provider, emergency room visit or hospitalization, use of systemic corticosteroids for asthma, or four or more asthma-related school absences. Adolescents were excluded if they had a significant developmental delay and/or severe psychiatric or medical conditions that precluded the completion of study procedures or confounded analyses.

To identify eligible adolescents, during the Fall 2019 and 2020 semesters, all adolescents in participating schools completed a brief screening survey in school. Trained study staff met individually with eligible students explaining the clinical trial and asking students additional questions regarding specific psychiatric or medical conditions that would result in study exclusion. Eligible adolescents who were interested in participating received consent/assent packages; parents/guardians signed consent forms, and adolescents signed assent forms. Demographic information was collected on the brief screening survey for the clinical trial and baseline measures were collected after consent/assent was complete. The Institutional Review Boards of Columbia University and the Medical University of South Carolina approved the study procedures.

### 2.2 Measures

#### 2.2.1 Socio-demographic and asthma characteristics

The participants reported their date of birth, which was used to calculate age, as well as their gender and race/ethnicity.

#### 2.2.2 Independent variable: depressive symptoms

The participants completed the widely used and validated Center for Epidemiological Studies Depression (CES-D) scale, a 20-item questionnaire that asks them to rate how often in a week they experienced symptoms associated with depression on a scale from 0 to 3 (0 = rarely or none of the time; 3 = most or almost all the time) (22, 23). The total scores could range from 0 to 60, with higher scores indicating greater depressive symptoms. The students with scores of 16 or greater were considered at risk for clinical depression. In our sample, the measure had good internal consistency (*α* = 0.86).

#### 2.2.3 Dependent variables

##### 2.2.3.1 Asthma self-management

To assess asthma self-management, we used three indices (24): (1) the *Asthma Prevention Index*; (2) *the Asthma Management Index*; and (3) the *Asthma Management Self-Efficacy Index*. The Asthma Prevention Index assesses what the participants do to prevent the onset of asthma symptoms. The participants in our study indicated the extent to which they take each of the nine steps to prevent the onset of symptoms (e.g., taking prescribed medication, avoiding triggers); we collapsed the positive responses to indicate whether the step was taken or not and computed a count of the total number of steps taken. The Asthma Management Index measures what participants do to manage acute symptoms. The adolescents indicated whether they took each of the seven steps or not (e.g., rest, took medication provided by their doctor). The total number of steps taken was summed. Both indices demonstrated good internal consistency in our sample (*α* = 0.82 and 0.79, respectively). Subsequently, the adolescents completed the 14-item Asthma Management Self-efficacy Index, which was used to assess their confidence in carrying out asthma self-management behaviors; each item was rated utilizing a six-point Likert scale (1 = very sure you could not, 6 = very sure you could). The index demonstrated good internal consistency (*α* = 0.83 for the current sample).

##### 2.2.3.2 Asthma control

The validated *Asthma Control Test* (ACT) (25, 26) was used to assess asthma control. The ACT is a patient self-administered five-point scale used to assess symptoms and activities to identify those with poorly controlled asthma. The scores range from 5 to 25, with higher scores indicating better-controlled asthma. Scores above 19 were considered well-controlled. The internal consistency among this sample was *α* = 0.68, which is considered an acceptable level of reliability (27).

### 2.3 Data analysis

We used linear regression to test associations between depressive symptoms, asthma self-efficacy, and asthma control. Because prevention and management were counted and their distribution followed approximately a Poisson distribution, we used Poisson regression to test associations between depressive symptoms and the number of steps taken to prevent asthma symptoms and manage existing symptoms. We controlled for gender and race/ethnicity because of the known associations to asthma prevalence, severity, and asthma-related quality of life (2, 4, 28–30). The models included school as a fixed effect. We used the case-wise deletion method to handle missing data. Those with and without missing data did not differ regarding demographic factors. All analyses were performed using SPSS 28.

## 3 Results

### 3.1 Participant characteristics

Baseline data from 200 adolescents from the trial were obtained; three cases were excluded due to missing predictor or outcome data (*n* = 2 for CES-D; *n* = 1 for self-management), resulting in an analytic sample of 197 adolescents. The age of the participants ranged from 14.47 to 19.92 years, with a mean age of 16.32 years. Most participants self-identified as female (68.53%). A majority self-identified as Black (62.43%). The remaining participants identified as White (19.80%) and Other (9.14%), which included Latino, Native American, or multiracial participants.

The descriptive statistics for the independent and dependent variables are detailed in Table 1. The mean CES-D score was 19.64; 61.4% of the participants were considered at risk for clinical depression (i.e., had scores of 16 or greater). On average, the participants took 5.39 steps to prevent the onset of symptoms and 4.80 steps to manage the symptoms once they began. Their mean asthma self-efficacy score was 4.33, and their mean ACT score was 18.42, with 61% having not well-controlled or very poorly controlled asthma.

**Table 1:** Participant characteristics.

Characteristic	*N* = 197
Age, years, mean (SD; range)^a^	16.32 (1.19; 14.47–19.92)
Sex, *n* (%)	
Female	135 (68.53)
Male	54 (27.41)
Not reported	8 (4.06)
Race/ethnicity, *n* (%)	
Black	123 (62.43)
White	39 (19.80)
Other (Latino, Native American, multiracial)	18 (9.14)
Not reported	17 (8.63)
Center for Epidemiological Studies Depression Scale	
Mean (SD; range)	19.74 (10.26; 1.00–49.00)
At risk for clinical depression^b^, *n* (%)	121 (61.42)
Number of prevention steps, mean (SD; range)	5.39 (2.53; 0–9.00)
Number of management steps, mean (SD; range)	4.80 (1.44; 0–7.00)
Asthma Self-Efficacy Index, mean (SD; range)	4.33 (0.78; 1.00–6.00)
Asthma Control Test	
Mean (SD; range)	18.42 (3.76; 8.00–25.00)
Well-controlled asthma^c^, *n* (%)	78 (39.60)
Not well-controlled asthma^c^, *n* (%)	75 (37.06)
Very poorly controlled asthma^c^, *n* (%)	46 (23.35)

^a^ Datapoint missing for one participant.

^b^ Defined as scores of 16 or greater (23).

^c^ Defined as follows: well controlled = scores of 20 and greater; not well controlled = scores of 19–16; very poorly controlled asthma = scores of 15 and lower (26).

### 3.2 Associations between depression, asthma self-management, and asthma control

As detailed in Table 2, depressive symptoms were significantly related to asthma control [*b* = −0.070; 95% confidence interval (CI) = −0.121 to −0.018], such that an increase in depressive symptoms was associated with worsening asthma control. Depressive symptoms were not related to asthma prevention, management, and self-efficacy.

**Table 2:** Associations between depressive symptoms, asthma self-management, and asthma control controlling for race/ethnicity and gender.

Variable	Prevention steps^a^	Management steps^a^	Asthma self-efficacy^b^	Asthma control^b^
	RR (95% CI)	RR (95% CI)	*β* (95% CI)	*β* (95% CI)
Depressive symptoms	1.00 (0.99–1.01)	1.00 (0.99–1.01)	−0.002 (−0.01 to 0.01)	−0.085** (**−0.14 to −0.03)**
Sex (Ref = female)	0.87 (0.74–1.02)	0.96 (0.80–1.12)	0.52 (−2.25 to 3.28)	−0.58 (−1.91 to 0.75)
Race/ethnicity (Ref = Black)				
White	0.63*** (**0.51–0.77)**	0.88 (0.72–1.08)	−0.14 (−0.46 to 0.18)	1.15 (−0.40 to v2.70)
Other	0.94 (0.74–1.17)	1.04 (0.82–1.31)	0.06 (−0.35 to 0.47)	−0.27 (−2.24 to 1.69)

CI, confidence interval; RR, relative risk.

^a^ Results from the Poisson regression model controlling for gender and race/ethnicity and using school as a fixed effect.

^b^ Results from the linear regression model controlling for gender and race/ethnicity and using school as a fixed effect.

**p < 0.01; ***p < 0.00.

## 4 Discussion

To our knowledge, this is the first study examining the relationship between depressive symptoms, asthma self-management, and asthma control in a rural adolescent population. Similar to studies with adolescents from Italy (7), individuals from the United States of ages 6–21 (8, 9), and adults from the United States (31, 32), we found an association between an increase in depressive symptoms and worsening asthma control. However, we did not find an association between depressive symptoms and the steps adolescents take to prevent asthma symptoms or manage existing symptoms nor between depressive symptoms and adolescents’ confidence to manage their asthma. The relationship between depressive symptoms and asthma self-management is not well-established, with some studies finding no associations and others finding associations, especially regarding trigger management (11). It is plausible that in this study we may not have adequately captured all the relevant aspects of asthma self-management that might be associated with depression. For example, while all three measures used have one or two items that assess trigger management, none enquire about communicating their symptoms or feelings about asthma to their healthcare provider or about how they interpret and react to symptoms—aspects of asthma self-management identified as being important to adolescents (33).

Several explanations might account for the association we observed between depressive symptoms and asthma control. With comorbid depression and asthma, there is an increase in inflammatory markers. One such marker is C-reactive protein (CRP), a potential biomarker of asthma control (34). CRP has been found to be elevated in children and adolescents with comorbid asthma and depression (35). In addition, autonomic nervous system (ANS) dysregulation may play a role. It has been proposed that depressed emotional states are associated with ANS dysregulation, which in turn increases disease severity (36). More specifically, the dysregulation in serotonin associated with depression has been shown to increase the secretion of Type 2 inflammatory cytokines commonly implicated in asthma (37, 38).

Alternatively, structural changes in the brain in patients with depression may contribute to worse asthma symptoms (39). Specifically, worse asthma symptoms have been associated with decreased homogeneity in the pallidum in patients with asthma and depression compared with those without depression (40). In addition, the anterior insula, an area of the brain associated with emotion processing, has been associated with increased inflammation in asthma (41). Also, depression has been found to be associated with a reduced bronchodilator response in adult patients with asthma (42). It is plausible this also occurs in adolescents.

Our findings may also be because asthma measures are self-reported, and adolescent responses may be impacted by depressive symptoms. Depressive symptoms have been found to influence how individuals perceive, process, and attend to information, with them often focusing on the negative aspects of a situation (43–45). Therefore, adolescents with asthma and comorbid depressive symptoms may perceive more asthma symptoms than their counterparts without depressive symptoms, resulting in their overreporting of symptoms (46, 47).

Our study is not without limitations. It is a secondary data analysis of cross-sectional data. As such, we were unable to include other variables that may be related to asthma or depressive symptoms (e.g., exposure to cigarette smoke, obesity, environmental factors). In addition, we were unable to examine causality or directionality, and our sample comprised mostly adolescents with poorly controlled asthma (i.e., signs and symptoms of asthma morbidity) who identified as female and Black; the youth were only from rural areas. Thus, generalizability to other populations is limited. Related to this is the fact that we had a relatively small number of adolescents in certain demographic groups (e.g., only 39 adolescents identified as White), which precluded our ability to test for moderation effects, which require larger sample sizes (48). Finally, our study did not include objective data like pulmonary function tests. However, poorly controlled asthma is not always reflected in pulmonary function tests, and patients with poorly controlled asthma may have normal pulmonary function tests (49).

This study is novel in examining associations between depressive symptoms, asthma self-management, and asthma control in adolescents. Given the association between asthma control and depressive symptoms found in this study, we recommend healthcare providers recognize the potential role of depressive symptoms in asthma. They may want to routinely screen for depression in their adolescents with asthma and, when warranted, offer referrals to psychosocial resources. We recommend future research include the use of objective measures and/or include multiple reporters, as well as more diverse samples. In addition, better study designs are needed that can establish causality (e.g., case controls) and examine the potentially bi-directional relationship between depression and asthma.

## Data Availability

The raw data supporting the conclusions of this article will be made available by the authors upon reasonable request.
